# How Are Healthcare Providers Conscientiously Objecting to Abortion in Australia? A Qualitative Study

**DOI:** 10.1111/psrh.70058

**Published:** 2026-02-11

**Authors:** Bronwen Merner, Casey M. Haining, Lindy Willmott, Julian Savulescu, Louise A. Keogh

**Affiliations:** ^1^ Melbourne School of Population and Global Health, University of Melbourne Parkville Victoria Australia; ^2^ Australian Centre for Health Law Research, Faculty of Business and Law, Queensland University of Technology Brisbane Queensland Australia; ^3^ Oxford Uehiro Centre for Practical Ethics, University of Oxford Oxford UK; ^4^ Centre for Biomedical Ethics, Yong Loo Lin School of Medicine, National University of Singapore Singapore Singapore; ^5^ Murdoch Children's Research Institute Parkville Victoria Australia; ^6^ University of Melbourne Parkville Victoria Australia

**Keywords:** abortion, conscience, health personnel, legislation, refusal to treat

## Abstract

**Introduction:**

Researchers have done limited empirical work to explore how healthcare providers are claiming conscientious objection to abortion care in Australia. Without this research, we cannot assess if existing mechanisms to regulate conscientious objection meet the needs of abortion seekers, abortion providers, and healthcare providers who conscientiously object to abortion care.

**Methodology:**

We conducted semi‐structured interviews with 41 interest‐holders (including healthcare providers who provided or conscientiously objected to abortion care) across Australia about conscientious objection to abortion care and its regulation. We analyzed the data using framework analysis.

**Results:**

We identified four themes describing how healthcare providers were claiming conscientious objection to abortion care. First, claims existed on a spectrum from “partial provision” to “refusal without referral.” When healthcare providers refused to provide an abortion, they did not always refer the abortion seeker to a willing provider or service. Second, claims of conscientious objection could change over time. Third, the relationship between religion and conscientious objection was not necessarily direct. Finally, some healthcare providers refused to provide abortion for reasons other than conscience.

**Conclusion:**

The findings demonstrated that conscientious objection provisions provided a flexible mechanism for healthcare providers to opt‐out of providing abortion care at different times, in different contexts, and for different reasons (including reasons other than conscience). Education and guidelines may improve healthcare providers' understandings and interpretations of conscientious objection provisions. Destigmatizing interventions may also reduce the number of healthcare providers who refuse to participate in abortion care for conscience‐based and non‐conscience‐based reasons.

## 1 Introduction

Abortion is a common healthcare procedure that is lawful in most countries [1]. Despite significant gains in decriminalizing abortion over the past 20 years, access to high quality abortion care remains elusive. A key reason for poor quality abortion care is the shortage of providers willing to provide abortion care [2]. Healthcare providers may refuse to provide abortion care for a range of reasons, including lack of training in abortion, stigma concerns, and institutional restrictions [3, 4, 5].

Conscientious objection is another reason healthcare providers may refuse to participate in abortion care. A provider engages in conscientious objection when he/she/they refuse to provide a legally and professionally accepted good or service that is within his/hers/their scope of practice for reasons of conscience [6]. This may include refusing to provide some or all abortions on conscience grounds. Conscience can be defined as the core subset of a person's moral beliefs that is integral to their self‐identity [6]. Many countries permit providers to refuse to participate in abortion care for reasons of conscience (unlike other reasons for non‐provision) [7].

In Australia, regulation seeks to balance a healthcare provider's right to conscientiously object with the right of an abortion seeker to access legally available health care [8]. In every Australian jurisdiction, legislative provisions permit healthcare providers with a conscientious objection to exempt themselves from participating in aspects of abortion care, in non‐emergency situations, subject to certain obligations [8]. The nature of these obligations vary across jurisdictions, but commonly include some form of disclosure requirement and referral requirement either an indirect referral through provision of information about available services or a direct referral to a non‐objecting healthcare provider or health service [8]. Other forms of regulation support conscientious objection provisions, such as conscientious objection policies of professional bodies (e.g., Australian Medical Association) and education about conscientious objection in undergraduate or postgraduate curricula [8, 9].

To date, limited empirical research has explored how regulation of conscientious objection to abortion care is working in Australia. In Victoria, studies have found that some healthcare providers with a conscientious objection did not comply with their referral obligations [3, 10, 11, 12]. In New South Wales, Lee and colleagues [13] found some pharmacists with a conscientious objection did not provide continuity of care when refusing to dispense medication abortion pills. Furthermore, a national study of conscientious objection among pharmacists (including, but not limited to, conscientious objection to abortion care) found that pharmacists who chose not to supply medications (including medication abortion pills) believed it was justifiable even if the refusal risked the patient not accessing treatment [14]. Collectively, these findings provide an emerging evidence base that healthcare providers' rights to object to abortion care are not being appropriately balanced with abortion seekers' rights to access high quality care. However, without an in‐depth, Australia‐wide study involving a range of interest‐holders (including clinicians and regulatory interest‐holders), we cannot determine how the current regulation of conscientious objection is working in practice. We use the term interest‐holder in preference to stakeholder given the latter term's colonialist roots [15].

To address this need, we conducted the Optimizing the Regulation of Conscientious Objection to Abortion research project. Our study was a national, qualitative research project running from 2021 to 2024, in which we explored how the regulation of conscientious objection to abortion care was operating across Australia. We also sought to identify areas for regulatory reform to appropriately balance the needs of people seeking abortion, abortion providers, and healthcare providers who conscientiously objected to abortion care.

In this article, we report on a subset of data from the ORCA study to address the following research question: How do a range of interest‐holders perceive healthcare providers are claiming conscientious objection to abortion care in Australia?

## 2 Methods

This study used semi‐structured interviews to explore the views of interest‐holders about how conscientious objection to abortion care was working in practice. The study received ethics approval from the University of Melbourne Human Research Ethics Committee.

### 2.1 Recruitment

We used a purposive sampling approach to recruit participants with a range of views on optimizing the regulation of conscientious objection to abortion care. These included interest‐holders from the following groups: healthcare providers who provided abortion, healthcare providers who self‐identified as conscientious objectors to abortion care, and a range of regulatory influencers including executives of health services that provided or refused to provide abortion care, pregnancy support services, professional colleges and unions, abortion seekers or people who have had abortions, politicians (both pro‐life and pro‐choice), government policymakers, medical insurers, and academics with expertise in conscientious objection to abortion care.

We identified potential participants and recruited through our professional networks, advertisements on social media, and early study participant referral. B.M. sent eligible participants an invitation letter and a copy of the consent form. All participants provided consent prior to participating. Most participants provided written consent by signing a consent form agreeing to participate. For participants providing verbal consent, B.M. read each statement from the consent form aloud and asked whether they consented. B.M. liaised with each participant to agree on a mutually convenient time and interview mode. Participants did not receive compensation for participation.

### 2.2 Data Collection

B.M. completed a semi‐structured interview with all participants in person, online, or over the telephone, according to participants' preferences. We chose semi‐structured interviews because they allowed us to explore participants' understandings, perceptions, and constructions of conscientious objection [16]. The interview guide (available on request) covered a range of topics, from participants' own experiences and impacts of conscientious objection to abortion care to their opinions about optimal regulation. Interviews were audio‐recorded and transcribed verbatim by a professional transcription company or B.M. and de‐identified prior to analysis.

### 2.3 Data Analysis

B.M. analyzed the interview data in NVivo [17] using framework analysis [18]. Framework analysis involved the following five stages: familiarization, identifying a thematic framework, indexing, charting, mapping, and interpretation [18]. As B.M. conducted the interviews, she was already familiar with the data and had started to identify potential themes during the data collection phase. B.M. initially constructed a thematic framework informed by the research's aims, issues raised by participants, and emerging themes. B.M. used the framework to index the data systematically.

The thematic framework included categories related to our domains of inquiry, such as experiences of conscientious objection, management of conscientious objection, impacts of conscientious objection, reasons for conscientious objection, protection of conscientious objection and optimal regulation of conscientious objection. Once B.M. indexed the data, she devised a chart to describe the data set as a whole. The chart combined the themes in the thematic framework with the research question and included headings that linked different themes; for example, a continuum of conscientious objection, reasons for not referring. At this stage, L.A.K. provided feedback on the thematic framework (during initial drafts of the manuscript) and B.M. amended accordingly. Finally, in the mapping and interpretation phase, B.M. revised the chart, with individual participants' experiences compared with patterns and connections identified. This included mapping the range of participants' perceptions and experiences of conscientious objection claims (e.g., the spectrum of conscientious objection), as well as identifying key concepts of how conscientious objection was being practiced (e.g., that conscientious objection is changeable, that the relationship between conscientious objection and religion is not direct and that there are claims of conscientious objection for non‐conscience‐based reasons). During this final stage, B.M. implemented feedback from all co‐authors on the coding framework to ensure the mapping and interpretation stage was robust.

### 2.4 Reflexivity

All investigators identified as pro‐choice and the declared aims of the project (as outlined in the Plain Language Statement) were to increase access to abortion as well as to protect the moral integrity of clinicians. We received feedback from a couple of participants that the pro‐choice position of the investigators as well as the study's aim to increase abortion access would dissuade conscientious objectors from participating in the study. We could have potentially mitigated this impact by having an investigator who identified as morally opposed to abortion care; however, this was not feasible within the timeframe of the project.

## 3 Results

### 3.1 Sample Characteristics

We recruited 41 participants for the study. Methods of recruitment included professional networks (*n* = 27), referral from existing participants (*n* = 9), and advertisements via newsletter or social media (*n* = 5).

#### 3.1.1 Recruitment of Healthcare Providers With a Conscientious Objection

Despite extensive efforts to recruit healthcare providers who identified as holding a conscientious objection to abortion care, we had a high non‐response rate among this population. We invited five organizations for healthcare providers with religious affiliations to advertise the study, of which one group agreed (resulting in one participant) and four did not respond. Of three pro‐life advocacy groups contacted, two responded but declined the invitation to participate. One of these groups, which provided permission to publish its reason for refusing, stated that the study's aim to benefit women by helping to improve provision and access to abortion was counter to the organization's aim to defend life from conception to its natural end. We also invited 11 clinicians who publicly identified via their practice website or media reports as conscientious objectors; one agreed to participate. We attempted to address the low representation of healthcare providers with a conscientious objection in the sample by recruiting three academics who had written about conscientious objection from a pro‐life perspective. We also recruited two politicians who self‐identified as pro‐life.

In total, B.M. conducted 37 interviews with 41 participants between December 2022 and January 2024. B.M. conducted most interviews with individual participants; however, she conducted four joint interviews on request. B.M. conducted 34 interviews via Zoom, two via phone, and one face‐to‐face. On average, interviews lasted 60 min.

The sample included 16 participants with a primary role of healthcare provider, including 12 who provided abortion and four who self‐identified as conscientious objectors to abortion care. Table 1 shows the characteristics of the healthcare providers.

**TABLE 1:** Characteristics of participating healthcare providers, including professional discipline and abortion provider/objector status (*n* = 16).

Professional discipline	Identified as an abortion provider (*n*)	Identified as a conscientious objector to abortion (*n*)	Total (*n*)
Obstetrics and gynecology	3	2	5
General practice	3	2	5
Midwifery	1	0	1
Sexual and reproductive health nursing	2	0	2
Pharmacy	2	0	2
Community liaison	1	0	1
	12	4	16

Table 2 shows the characteristics of the whole sample (*n* = 41).

**TABLE 2:** Characteristics of all participants, including primary role and position on abortion (*n* = 41).

Primary role				
Healthcare providers and services	Total	Provider of abortion services	Self‐identified individual CO or institutional objector	Neutral
Healthcare provider	16	12	4	0
Executive of health service/pregnancy support service	7	6	1	0
	23	18	5	0

Advocates	Total	Self‐identified as pro‐choice	Self‐identified as pro‐life	Did not self‐identify as pro‐life or pro‐choice
Advocacy group	1	1	0	0
Politician	4	2	2	0
Academic	3	0	3	0
	8	3	5	0

Other regulators				
Government policymaker	7	0	0	7
Professional college or union	2	0	0	2
Medical insurer	1	0	0	1
	10	0	0	10
Total	41	21	10	10

### 3.2 Themes

Given the small number of participants who identified as objectors, the data set combines objectors' own descriptions and experiences of holding an objection, as well as clinicians' and other regulatory influencers' perceptions or experiences of conscientious objection in the health system. We allowed participants to define conscientious objection, rather than pre‐defining it, to allow us to explore what the concept meant to them.

We identified four main themes that encompassed how interest‐holders perceived conscientious objection to abortion care was being claimed by healthcare providers in Australia: (1) Claims of conscientious objection existed on a spectrum; (2) Claims of conscientious objection could change over time; (3) The relationship between religion and conscientious objection was not necessarily direct; and (4) Some healthcare providers did not provide abortion for reasons other than conscience.

#### 3.2.1 Claims of Conscientious Objection Existed on a Spectrum

Rather than being solely an objector or a provider, healthcare providers could straddle positions as both provider and objector. Practitioners who provided at times and refused to provide at other times existed at the “partial provision” end of the spectrum. They maintained their moral integrity by imposing personal limits on provision. Providers occupying the middle of the spectrum (refusal with referral) objected but were willing to refer the patient onwards—at least sometimes—because they separated their personal views from their professional obligations. Practitioners at the “refusal without referral” end of the spectrum refused to provide but also refused to refer on the grounds that the act of referral would violate their moral integrity.

##### 3.2.1.1 Partial Provision

For healthcare providers at the “partial provision” end of the spectrum, some held conscientious objections to specific types of abortion care. For example, they were comfortable providing abortion care for all reasons within the first trimester but imposed personal limits on second and third trimester abortion care:

And then doctors who won't work past certain gestation limits, yeah. And I don't know if they would describe themselves as conscientious objectors, but they have set for themselves limits around the services that they would provide. In one instance when I asked the doctor what that was about, I don't think it was necessarily about their training and competency, it was their discomfort about working past a certain gestation limit.

—Manager, health or support service, Victoria

Some healthcare providers would perform abortions, including at advanced gestations, but objected in cases where they did not perceive there was a compelling reason to do so. For some, these reasons could be wide‐ranging, but they needed to perceive the reason/s as sufficiently serious to warrant the abortion. For example, a Queensland maternal–fetal medicine specialist (MFM) identified “extremes of family violence” or a “maternal suicide attempt” as sufficient grounds for an abortion at 24 to 28 weeks.

In contrast, some healthcare providers objected to performing most abortions, with only a very limited set of exceptions. For example, a Victorian obstetrician‐gynecologist, who self‐identified as an objector, would only perform abortions for severe fetal abnormalities or severe mental illness.

##### 3.2.1.2 Refusal With Referral

For healthcare providers with objections who refused to provide abortion at all, some would refer abortion seekers appropriately to relevant services. These providers separated their personal beliefs from their professional obligations. For example, a female general practitioner in New South Wales would not prescribe medication abortion pills due to her religious views but would “help [abortion seekers] access whatever care they need” because she did not want her own beliefs to interfere with pregnant people's choices. In another example, a Queensland nurse recalled a colleague who objected to participating in abortion care and felt a professional obligation to refer to ensure culturally safe abortion services were available to pregnant people in her community:

[The provider] still doesn't believe in abortion, she doesn't like it but she is the main person supporting First Nations women to get abortions in [location] because she said “I need to do this for my people because I need to get them the best health care that they can, because actually me… not providing abortions doesn't stop them getting abortions, it actually means that they're getting culturally unsafe abortions.”

However, some participants (who were not self‐identified objectors) reported that objecting doctors did not refer consistently. Instead, they referred some patients and not others. A participant from a medical indemnity insurance agency explained:

Even if [a doctor] has a conscientious objection, they may be happy to do that [abortion] referral, just as part of that … ongoing doctor/patient relationship on the understanding that I can't help you for this aspect of your care, but I'd like to remain your doctor and come back and we'll continue to have a doctor/patient relationship… I guess as opposed to a situation if it's a walk‐in clinic or someone you don't know, they're just simply there to get a prescription for medication. And then you're saying, “Well, sorry, I can't give that.”

##### 3.2.1.3 Refusal Without Referral

At the refusal without referral end of the spectrum, some healthcare providers who objected would not refer for abortions, even when legally required to do so. Interview participants from pregnancy support services reported GPs commonly responded to abortion requests by saying they did not agree with abortion and would not help the patient to access one.

One reason for not providing a referral was that healthcare providers with objections felt the act of referral meant they were morally complicit in the abortion. For example, a male GP from Western Australia, who refused to provide abortion care for religious reasons, believed that referral would violate his moral integrity:

I guess it comes down to why you oppose abortion. For me … my actions are informed by my Christian faith, and so … I believe that life begins at conception and that is an absolute and non‐negotiable, so I'm not going to have anything to do with it.

He went on to describe how his position compared to others with a conscientious objection:

They may not oppose it quite as strongly as I do, I guess you might say, and so they would be more comfortable that, “I don't want anything to do with this, but I appreciate that it's your decision, and so you can go and see Dr Such and Such down the road.”

Illustrative of the complexity of maintaining personal moral integrity when opposed to abortion care, a male Ob/Gyn felt morally comfortable performing abortions for severe fetal anomalies or significant maternal illness. However, he felt morally complicit providing a direct referral for abortion care in other circumstances:

If you say to someone, “Look, there's an organisation called Marie Stopes, or you can Google it,” it's not like you're taking them to the place and giving them an injection … But to actually give them a written paper or to say, “Here doctor, here is Mrs So and So. Please give her an abortion.” That's a different thing.

Some participants conveyed that abortion services were easy to find and access, with or without a referral. This assumption could lead some healthcare providers to feel that refusing to provide a referral would not result in harm to the abortion seeker:

The organisations that [provide abortion care] don't require referrals. Patients can refer themselves, and they don't keep themselves secret … I think they're easy to find. And people have Google and it's not that difficult to find [abortion services].

—GP, self‐identified objector, New South Wales

##### 3.2.1.4 Participation in Pre‐ and Post‐Abortion Care

Healthcare providers with a conscientious objection who refused to provide abortion also had different levels of comfort participating in pre‐ and post‐abortion care. Some healthcare providers would organize all necessary tests and after care, whereas others would not. Also, providing misoprostol to treat an incomplete abortion was acceptable for some but not all:

If we take this group of doctors who didn't want to provide [medication] abortion, some were happy to provide follow up treatment for an incomplete abortion, for instance, by giving extra misoprostol, but some weren't. So, they had lots of those different levels of what people would actually feel comfortable doing.

—Manager, health or support service, New South Wales

#### 3.2.2 Claims of Conscientious Objection Could Change Over Time

Healthcare providers' beliefs or practices relating to abortion care could change over time, which influenced whether they claimed a conscientious objection. This could be in response to personal or professional experiences. Changes in regulation, such as decriminalization of abortion, also shaped claims of conscientious objection.

A couple of participants recounted experiences of healthcare providers who had changed their core views on participating in abortion care due to personal or professional experiences. This shifting view suggests conscientious objection is flexible rather than fixed. A clinical manager of a health service in Victoria discussed their experiences with a doctor who subsequently became a provider:

I can remember a young woman of African descent who grew up in the faith of Islam and struggled a little bit and she was one of [the Ob/Gyn] trainees, and it was when she started to look after [Muslim] women requesting abortion care that she started to make a bit of a shift herself.

In the opposite direction, one participant reported an experience with a GP obstetrician who had provided abortion and became an objector following reflection about their views on the beginning of life.

Claims of conscientious objection could also increase when significant regulatory changes occurred, such as the decriminalization of abortion. A MFM reported that recent decriminalization in his state (Queensland) shifted decisions about third trimester abortion onto abortion providers. The new law provided for terminations at later gestations provided two doctors approved the termination. The MFM recounted a request for a third trimester abortion where he considered the pregnant person did not have “mitigating” circumstances:

There were no practitioners at the hospital who said that they would do it. Despite the fact that we're a very pro‐choice hospital and we offer termination of pregnancy services. I guess … that's an example of how the current legislation is now really leading to some practitioners having to exercise their own form of conscientious objection. It's not that they're against termination of pregnancy, it's that they're against termination of pregnancy at any stage for any reason.

Similarly, the loosening of restrictions on prescribing medication abortion pills led to ethical conflict for some clinicians. For example, prior to medication abortion pills being widely available in Australia, the female GP who objected to providing abortion care could work in sexual and reproductive health without feeling concerned about violating her moral integrity:

… in the beginning, we didn't have access [to medication abortion pills] anyway. It was only very few clinics that offered it. So [my role] never tended to be in conflict with anything that I believed in terms of my own religious background.

Another example of a regulatory change potentially uncovering conscientious objection was the removal of mandatory training required to prescribe medication abortion pills. Two participants, who refused to provide medication abortion pills for religious reasons, commented that because clinicians could no longer decline provision by stating they had not undertaken the necessary training, they were now having to declare their objection to patients.

#### 3.2.3 The Relationship Between Religion and Claims of Conscientious Objection Was Not Necessarily Direct

The four healthcare providers in the sample who self‐identified as objectors all claimed a conscientious objection to abortion care on religious grounds. Additionally, most of the other participants perceived that religious beliefs were the main reason that healthcare providers claimed conscientious objection. However, being religious did not necessarily correlate with a refusal to participate in abortion care. This could be because not all religions prohibit abortion or people interpret religious teachings differently. As a female Victorian Ob/Gyn, who identified as a conscientious objector, described:

I'm aware that there are religious people who still feel that the God, or the creator or whoever, put certain skills into humans' hands to prevent suffering. Some people will interpret that as if abortion relieves suffering, well then that is in line with what God thinks. But other religions, say Catholic[ism], traditionally doesn't look at it like that.

A few participants stated they knew of religious healthcare providers who were providing abortion. For example, a health service manager in New South Wales stated:

I've come across people where they've worked through their collective religious beliefs to be able to still provide sometimes late term abortions. And often in that situation, they will declare, “I'm a Catholic,” “I'm a Greek Orthodox person, but I've made this decision to provide these services.”

This suggests that while some religious doctors do claim an objection for reasons of conscience, others do provide abortion care.

#### 3.2.4 Some Healthcare Providers Did Not Provide Abortion for Reasons Other Than Conscience

Participants perceived there were a range of reasons not related to conscience that influenced healthcare providers to refuse to participate in abortion provision. The two key reasons were lacking the knowledge, training, or need to provide abortion and abortion stigma and discrimination. Table 3 summarizes these reasons.

**TABLE 3:** Non‐conscience‐based reasons for not providing abortion, according to participants.

Reason	Sub‐themes
Lacking the knowledge, support, or need to provide abortion	Lacking knowledge about Abortion law CO provisions Referral pathways
	Feeling unprepared or lacking confidence and support to provide medication abortion
	Perceived lack of need for abortion services
Abortion stigma and discrimination	Perceiving abortion as: Outside mainstream health care Unpleasant or onerous
	Holding discriminatory attitudes for example, misogyny, racism

We elaborate each non‐conscience‐based reason for refusal to participate in abortion care below.

##### 3.2.4.1 Lacking the Knowledge, Support, or Need to Provide Abortion

Healthcare providers who lacked knowledge of abortion law (including legality and gestational limits) could refuse to participate in abortion care. However, addressing these knowledge gaps could lead to change. One participant recounted working with a male pharmacist who refused to dispense medication abortion pills because he thought abortion was illegal in Australia. Once the participant explained abortion was legal, the pharmacist started dispensing. Several participants recounted that some providers did not refer because of a lack of knowledge and training:

… Some of the feedback we're getting is it's not necessarily conscientious objection; it's lack of knowledge and training … So the result for the patient might be the doctor can't assist them. But that may not be because of conscientious objection.

—Medico‐legal insurer

Lack of knowledge of conscientious objection obligations could also contribute to non‐provision. When asked about the referral requirement for healthcare providers with objections, some participants in the sample (both self‐identified providers and objectors) did not know there was a legal obligation to refer. This suggests there could be a general lack of awareness of conscientious objection provisions among healthcare providers. Additionally, lack of knowledge could also extend to poor or absent knowledge of appropriate referral pathways for abortion care.

Lack of training and support to provide abortion care could negatively impact a healthcare provider's views on providing abortion care. When healthcare services wanted to establish new abortion services or broaden the abortion services they provided, some existing staff were reluctant to start providing abortion care because they felt unprepared:

Either it's not what they signed up for when they became a midwife or [decided to] work in maternity care. Or they don't feel like they have the training and experience in that care to be able to provide it. They're very senior otherwise but feel like they're completely unprepared or don't want to appear like a novice. So, I think all of those things are backed up in what we call conscientious objection.

—Government policymaker, 18+ years of experience

In contrast, in some practice settings, GPs did not provide medication abortion pills due to a lack of need. For example, in larger metropolitan practices, some GPs (who did not hold objections) did not provide abortion because the practice already included a medication abortion provider and there were only a few requests for medication abortion each year.

##### 3.2.4.2 Stigma and Discrimination

The stigma of abortion and abortion provision prevented, or at least limited, healthcare providers from engaging in abortion care. Stigma could manifest as conscientious objection because claiming conscientious objection provided a mechanism for refusing to participate in abortion care:

… if you can choose not to participate in a form of stigmatised health care by just saying “I'm a conscientious objector” and that's it, unless you actually care about people's access to abortion, it's a pretty easy way to opt out.

—Manager, health or support service, Queensland

Some participating abortion providers were concerned that healthcare providers declined to participate in abortion care because they did not view abortion as mainstream health care. Individual decisions to refuse to participate in abortion care could reflect the broader social context of stigmatization of abortion services, and fear of potential inundation of abortion cases:

I think it's easy to demonise individuals who one week will do it as a favour, but don't want to do it regularly. Like it's not just about the individual perhaps not stepping up. I think there's obviously a context in which they're making these choices. And it's about stigmatisation of this service. And also, a fear of being seen as the abortion provider for that hospital and then being overloaded with all of that work.

—MFM, New South Wales

Finally, workplace culture could affect whether healthcare providers participated in abortion care. In a medical practice, senior clinicians who objected to abortion care influenced other clinicians not to provide:

… we've got a limited supply of people able to provide those abortion services and then they're being stigmatised by … other people in the practice who conscientiously object and so basically impose that requirement on everybody in a practice.

—Government policymaker, 15+ years' experience

Negative perceptions of the abortion procedure as “icky, messy, too much trouble” (Manager, health service, New South Wales) could also affect providers' willingness to provide. Discomfort with abortion care could reduce a provider's motivation to seek information about local referral pathways that would help an abortion seeker to access care elsewhere:

I think that sometimes other types of objection to abortion, like “It's a bit too hard for me” “I don't really know what to do” gets bundled in with conscientious objection every now and again, because that's the easy reason to say why I don't do it.

—Manager, health or pregnancy support service, Victoria

A couple of participants believed that healthcare providers used conscientious objection as an excuse for discriminatory treatment of people seeking abortion. Discriminatory attitudes included those related to gender, whereby providers refused to participate in abortion because they believed it was women's role to bear children. A government policymaker with over 15 years' experience reported that healthcare providers had used conscientious objection as an excuse for racial discrimination:

And in a very tight … market around the provision of health services, and we see it with some of our Aboriginal community members, we see it with some of our Muslim community members, that there's various reasons why people then say, “I can't provide services to you,” allegedly because of some conscientious objection. And it's like, “I bet if I asked you to quote me a bit of the Book of Paul, you wouldn't be able to do it.”

## 4 Discussion

Our findings provide a nuanced account of how healthcare providers are claiming conscientious objections to abortion care in Australia. We found that conscientious objection to abortion care and abortion provision were not exclusive, binary positions, that providers' claims of conscientious objection could change, and that the link between religion and conscientious objection was not necessarily direct. Our analysis also demonstrated that, for some, refusing to provide abortion care was not related to conscience at all. The finding that a healthcare provider's conscientious objection can change is supported by previous research [19]. Other studies have also found that participation in abortion may be perceived differently by different objectors [19, 20, 21]. These findings suggest that ongoing dialogue and negotiation with providers who object about the tasks they *do* feel comfortable performing could help lessen the burden on providers who do not object [19]. As well, institutions that require notification of a health provider's conscientious objection should encourage notifications at periodic intervals rather than assuming a notification on commencement is enduring.

Previous authors have also found a continuum between partial provision and total objection [22, 23, 24]. Our findings showed that context played a significant role in providers' moral decision‐making about whether to provide abortion. In a previous study, Freeman and colleagues [24] described how the contextual factors surrounding an abortion served as tipping points for both providers and non‐providers about the moral acceptability of an abortion. This meant that decisions to provide were more complex than whether a provider perceived abortion as morally wrong [24]. Our research showed that, for providers who otherwise provided abortion, the intersection of later gestational age without a perceived compelling reason for the abortion could be enough to tip into non‐provision. In contrast, for providers who did not routinely provide abortion, medical reasons such as severe fetal anomalies or maternal health concerns could tip them towards provision. These findings mirror previous research conducted on abortion in Australia [25, 26]. Muller and colleagues [27] have argued that a provider's personal norms became particularly important in decision‐making when he/she/they are working in challenging policy environments, such as abortion. In these environments, when there is a lack of guidance, managerial communication and collegial support, providers use their personal values to decide who receives a service and how it is provided [27]. This suggests that optimizing the regulation of conscientious objection to abortion care will require improved education, guidance, and support for healthcare providers about abortion care to reduce their reliance on personal norms for decision‐making [27].

Consistent with previous Australian research, the study showed that healthcare providers with conscientious objections did not always refer abortion seekers for abortion, despite a legal obligation to do so [3, 10, 11, 12]. Like provision, context could also affect the likelihood of referral with reports that some providers were willing to refer regular patients for abortion care but not new patients. Although some refused to refer for moral reasons, other reasons may be more amenable to policy and education interventions. Echoing previous calls in the literature, easy‐to‐access guidelines may assist those who are unfamiliar with conscientious objection provisions and abortion legislation to comply [3, 14, 20]. Education about the risks of non‐referral, including the obstacles abortion seekers face in accessing abortion, may also help. Providers may also benefit from service models (such as 1800 My Options in Victoria, Australia) that provide readily accessible information and education about local abortion referral pathways [28]. Further to this, Fiala and Arthur recommend medical associations encourage and incentivize new abortion providers, especially outside major cities, by providing, for example, additional pay and benefits [29]. They also suggest improving complaints processes for patients by allowing them to complain about denial of abortion care without the offending doctor knowing their identity [29].

Our findings also demonstrated that law reform resulting in increased abortion access, such as decriminalizing abortion or introducing medication abortion, may have unintended consequences [30]. Some healthcare providers may adjust to the changes by imposing additional personal limits on provision. Other providers who become eligible to provide may choose to opt out. Interventions to reduce abortion stigma, such as Values Clarification Training or Providers Share Workshops, may support clinicians through regulatory changes [31, 32]. Such interventions could also assist those who do not currently provide abortion for reasons other than conscience or people who partially object to abortion in specific circumstances. Increasing abortion training for healthcare providers in reproductive health care also helps to normalize abortion care as health care [33].

Finally, the results showed that some healthcare providers claimed conscientious objection because of abortion stigma or discriminatory attitudes towards patients requesting abortion. This is consistent with previous research that individual conscientious objection claims can be rooted in or mask political, social, or other factors [34]. As conscientious objection protections are enshrined in legislation in Australia (as well as other countries), they should be considered a macro‐level factor in (re)producing abortion stigma [35]. However, the complex interplay between conscientious objection and abortion stigma requires further exploration [34, 35].

### 4.1 Limitations

Our study has two key limitations. First, abortion seekers were not included in the study. While we did invite an interest‐holder group representing people who have had abortions to participate, we did not receive a response. Understanding the perspective of abortion seekers about conscientious objection would have enriched our exploration of the current regulatory system, particularly its impacts on their access to abortion care. Second, only a small number of healthcare providers who identified as conscientious objectors agreed to participate in our study, despite sustained efforts to recruit this cohort. It is likely that many objectors who agreed to participate represented more moderate positions in their management of conscientious objection than those who were unwilling to participate. As such, the objectors' perspectives included in this study may be skewed towards those who are more mindful of their professional and legal obligations. Objectors who hold more extreme views and therefore engage in more extreme behaviors, such as being judgmental to patients or deliberately providing patients with misinformation, may have been less likely to participate in our study.

## 5 Conclusion

In this study, we described how current regulation of conscientious objection to abortion care is working in Australia and identified areas for reform to better meet the needs of abortion seekers, abortion providers and providers claiming a conscientious objection to abortion care. We recommended that healthcare providers require education about conscientious objection, including the obligations placed on a healthcare provider who refuses to participate in some or all abortions. Furthermore, given conscientious objection may change, institutions should encourage objectors to provide notifications at periodic intervals rather than assuming a notification on commencement is enduring. Finally, the use of destigmatizing interventions, such as Values Clarification Training, may help healthcare providers to move from non‐provision to provision.

## Funding

This work was supported by the Australian Research Council, DP210102916.

## Conflicts of Interest

J.S. is a Partner Investigator on an Australian Research Council (Grant number LP190100841) which involves industry partnership from Illumina. He does not personally receive any funds from Illumina. J.S. is a Bioethics Committee consultant for Bayer. The other authors declare no conflicts of interest.

## Data Availability

The authors have nothing to report.
